# Attenuation of Flightless I Increases Human Pericyte Proliferation, Migration and Angiogenic Functions and Improves Healing in Murine Diabetic Wounds

**DOI:** 10.3390/ijms21165599

**Published:** 2020-08-05

**Authors:** Hannah M Thomas, Parinaz Ahangar, Benjamin R Hofma, Xanthe L Strudwick, Robert Fitridge, Stuart J Mills, Allison J Cowin

**Affiliations:** 1 Future Industries Institute, University of South Australia, Adelaide 5000, Australia; hannah.mary.milroy.thomas@gmail.com (H.M.T.); parinaz.ahangar@mymail.unisa.edu.au (P.A.); Ben.Hofma@HRI.org.au (B.R.H.); xanthe.strudwick@unisa.edu.au (X.L.S.); stuart.mills@unisa.edu.au (S.J.M.); 2 Clinical and Health Sciences, University of South Australia, Adelaide 5000, Australia; 3 Cell Therapy Manufacturing Cooperative Research Centre, Adelaide 5000, Australia; 4 Faculty of Health and Medical Sciences, University of Adelaide, Adelaide 5000, Australia; robert.fitridge@adelaide.edu.au

**Keywords:** pericytes, diabetes, flightless I, angiogenesis, inflammation, wound healing

## Abstract

Pericytes are peri-vascular mural cells which have an important role in the homeostatic regulation of inflammatory and angiogenic processes. Flightless I (Flii) is a cytoskeletal protein involved in regulating cellular functions, but its involvement in pericyte activities during wound healing is unknown. Exacerbated inflammation and reduced angiogenesis are hallmarks of impaired diabetic healing responses, and strategies aimed at regulating these processes are vital for improving healing outcomes. To determine the effect of altering Flii expression on pericyte function, in vitro and in vivo studies were performed to assess the effect on healing, inflammation and angiogenesis in diabetic wounds. Here, we demonstrated that human diabetic wounds display upregulated expression of the Flii protein in conjunction with a depletion in the number of platelet derived growth factor receptor β (PDGFRβ) +/ neural glial antigen 2 (NG2) + pericytes present in the dermis. Human pericytes were found to be positive for Flii and attenuating its expression in vitro through siRNA knockdown led to enhanced proliferation, migration and angiogenic functions. Genetic knockdown of *Flii* in a streptozotocin-induced murine model of diabetes led to increased numbers of pericytes within the wound. This was associated with dampened inflammation, an increased rate of angiogenic repair and improved wound healing. Our findings show that Flii expression directly impacts pericyte functions, including proliferation, motility and angiogenic responses. This suggests that Flii regulation of pericyte function may be in part responsible for the changes in pericyte-related processes observed in diabetic wounds.

## 1. Introduction

Diabetes is a debilitating disease which manifests in many pathologies including retinopathy, neuropathy and delayed cutaneous wound repair [1]. Impaired healing often leads to the development of chronic wounds in diabetic patients, the most common type being diabetic foot ulcers (DFUs) [2]. DFUs are characterised by poor vascularisation and an exacerbated inflammatory response which fails to resolve appropriately [3]. 

Pericytes are peri-vascular mesenchymal stem cell (MSC)-like cells involved in the regulation of both vascularisation and inflammation [4,5]. In diabetic patients, many tissues exhibit a decrease in pericyte populations, and this reduction has been implicated in the progression of some diabetic pathologies [6]. Given that pericyte depletion is observed in the skeletal muscle of diabetic feet [7], it is possible that pericytes may also play a role in contributing to the delayed cutaneous healing experienced by diabetic patients. 

Flightless I (Flii) is a member of the gelsolin family of cytoskeletal proteins that regulate actin by severing pre-existing filaments and/or capping filament ends to enable filament reassembly into new cytoskeletal structures. Flii is involved in numerous cellular activities including regulating transcription via co-activation of nuclear hormone receptors [8,9] and regulation of β-catenin-dependent transcription. Flii contains a leucine-rich repeat region (LRR) which allows this protein to play an additional role in the mediation of cell signalling events [10]. Studies have shown that Flii acts as a negative regulator of wound healing [11]. Genetic knockdown of *Flii* leads to enhanced proliferation and migration of keratinocytes and fibroblasts, resulting in accelerated re-epithelialisation and contraction of acute wounds [12]. Flii attenuation has been explored as a therapeutic approach to repair, and treatment of wounds with a Flii neutralising antibody (FnAb) improves healing in murine and porcine models of acute cutaneous repair [12,13].

We have previously found that Flii negatively impacts healing in a streptozotocin (STZ)-induced murine model of type 1 diabetes [14]. Flii-attenuated diabetic wounds exhibit enhanced expression of some angiogenic markers, however the mechanisms leading to this have not been identified. In this study, the STZ-induced model of type 1 diabetes is again used as treated mice exhibit symptoms of human type 1 diabetes with chronic pancreatic islet inflammation, insulin deficiency and impaired wound healing [15]. Given the importance of pericyte participation for normal vascular repair, and the lack of pericytes observed in human diabetic patients, we aimed to investigate whether altered pericyte function underpins Flii effects on diabetic wound healing.

## 2. Results

### 2.1. Flightless I Expression Impacts Human Pericyte Function

**Figure 1:**
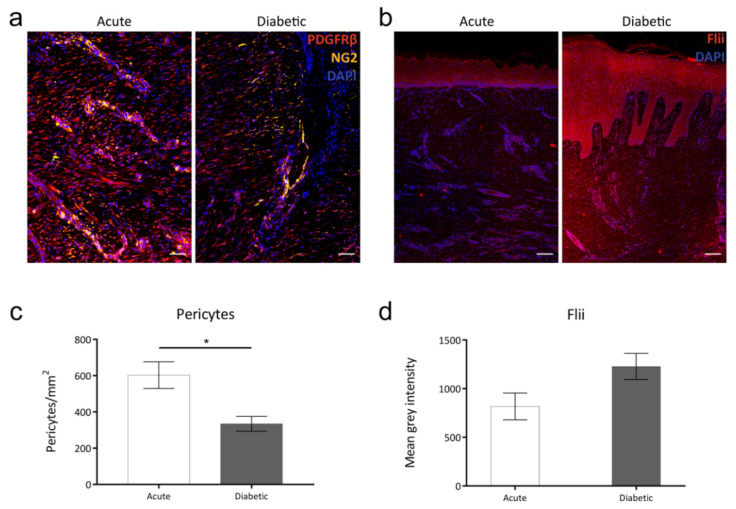
(**a**) Dual Platelet derived growth factor receptor β (PDGFRβ)/neural glial antigen 2 (NG2) pericyte staining in 4 μm sections of paraffin-embedded acute and diabetic human wounds, scale bar = 50 μm. (**b**) Immunohistochemical detection of Flightless I (Flii) in corresponding 4 μm sections of paraffin-embedded acute and diabetic human wounds, scale bar = 100 μm. Composite images captured at 20× objective. Graphical data of (**c**) the number of pericytes per mm^2^ within acute and diabetic human wounds and (**d**) the expression levels of Flii in acute and diabetic human wounds. * = *p* < 0.05.

Platelet derived growth factor receptor β (PDGFRβ)/neural glial antigen 2 (NG2) dual staining was carried out in 4 μm sections of paraffin-embedded human samples of acute wounds and diabetic wounds. Wound-resident PDGFRβ+/NG2+ pericytes were counted and normalised to wound area for each wound. Pericytes were observed in all wounds, however pericyte numbers were significantly lower in diabetic than in acute wound samples (Figure 1a,c). Immunohistochemical analysis of Flii expression in human wound samples showed a strong trend towards increased Flii expression in diabetic wounds when compared to acute wounds, although this difference was not statistically significant (*p* = 0.0724). As such, pericyte numbers in the human wounds examined displayed an inverse relationship with Flii expression, supporting the notion that Flii expression may have a negative impact on wound healing by affecting the functionality and survival of wound-resident pericytes during diabetic healing (Figure 1b,d).

**Figure 2:**
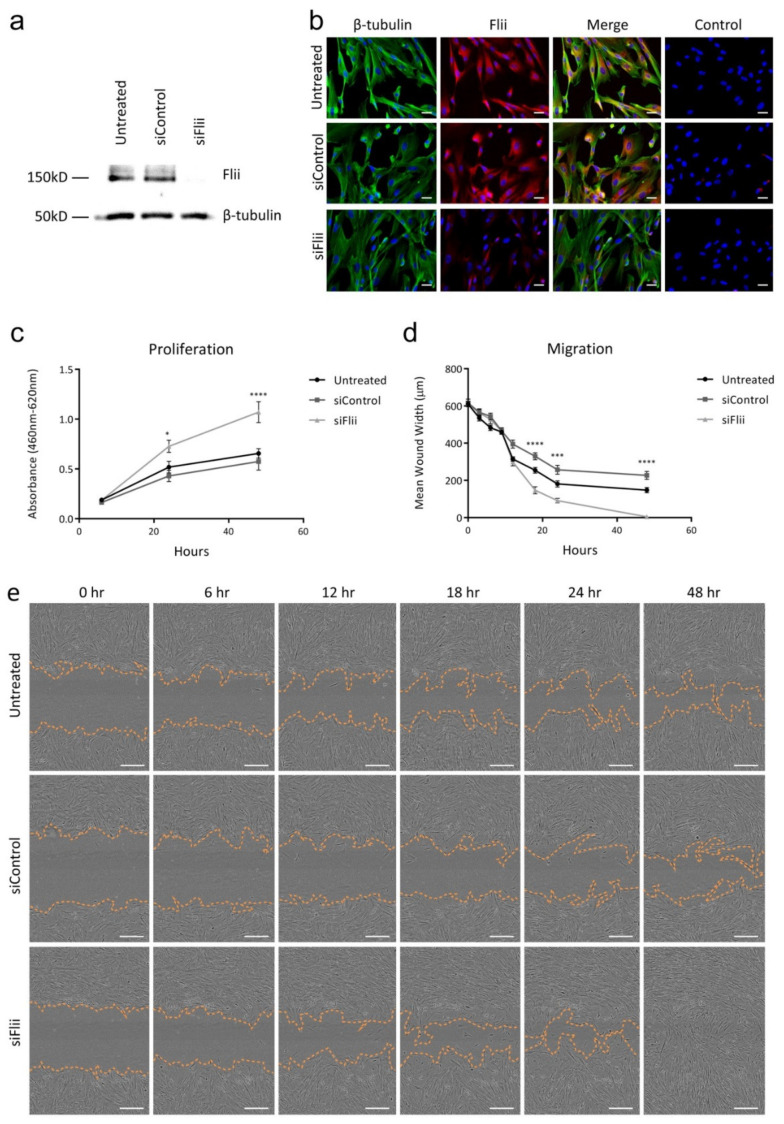
(**a**) *Flii* knockdown by siRNA as shown by Western Blotting of untreated, siControl- and siFlii-treated human pericytes from placenta (hPC-PL) cell lysates 48 h after knockdown. (**b**) *Flii* knockdown by siRNA as shown by immunocytochemical staining of untreated, siControl- and siFlii-treated hPC-PL cells 48 h after knockdown. No primary antibody-treated hPC-PL cells are shown as control. Images taken at 40× objective, scale bar = 20 μm. (**c**) Effect of *Flii* knockdown on hPC-PL proliferation in a WST1 proliferation assay. Data displayed as mean ± SEM, *n* = 6. Results are representative of three independently conducted experiments. (**d**) Effect of *Flii* knockdown on hPC-PL migration in a scratch wound assay. Data displayed as mean ± SEM, *n* = 6. Results are representative of three independently conducted experiments. (**e**) Images representative of untreated, siControl-treated and siFlii-treated hPC-PL scratch wounds at 0, 6, 12, 18, 24 and 48 h. Images taken at 4× objective, scale bar = 300 μm. Statistical significance was calculated using a two-way analysis of variance (ANOVA) where * = *p* < 0.05, *** = *p* < 0.001, **** = *p* < 0.0001.

In order to directly investigate the impact of Flii expression on pericyte function, it was necessary to knock down Flii expression in human pericytes for use in functional assays. SiRNA knockdown of *Flii* was carried out in pericytes. Immunocytochemical staining and Western blotting of lysates from untreated, siControl-treated and siFlii-treated human pericytes from placenta (hPC-PL) indicated that human pericytes (hPC-PL) expressed Flii in culture, and siRNA knockdown of Flii induced markedly decreased levels of Flii protein expression after 48 h (Figure 2a,b). Proliferation and migration rates of untreated, siControl-treated and siFlii-treated hPC-PL were assessed with a WST1 assay and scratch assay, respectively. Attenuation of Flii led to accelerated proliferation (Figure 2c) and migration (Figure 2d,e) of pericytes when compared to untreated and siControl-treated cells (*n* = 6).

### 2.2. Flii Expression and Diabetic Status both Negatively Impact Dermal Pericyte Numbers In Vivo

**Figure 3:**
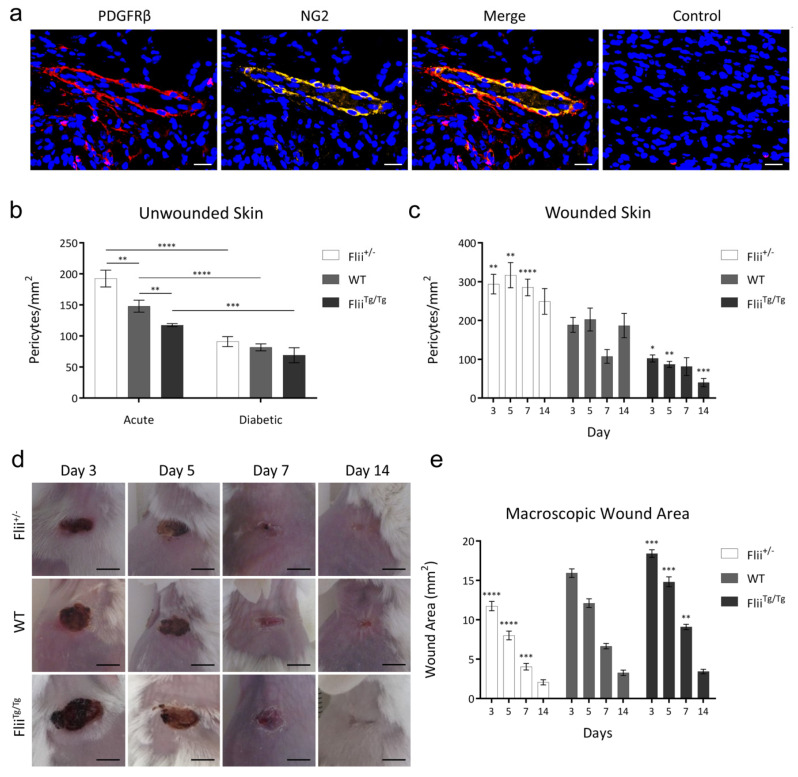
(**a**) Dual PDGFRβ/NG2 pericyte staining in 4 μm sections of paraffin-embedded murine tissue. No primary antibody-stained section is shown as control. Composite images taken at 20× objective, scale bar = 20 μm. (**b**) PDGFRβ/NG2^+^ pericyte numbers in acute and diabetic unwounded skin of *Flii*^+/−^, WT and *Flii*^Tg/Tg^ wounds. Data displayed as mean ± SEM, *n* = 6. (**c**) PDGFRβ/NG2^+^ pericyte numbers in diabetic *Flii*^+/−^, WT and *Flii*^Tg/Tg^ wounds. Data displayed as mean ± SEM, *n* = 8. (**d**) *Flii*^+/−^, WT and *Flii*^Tg/Tg^ diabetic excisional wounds collected at 3, 5, 7 and 14 days post-injury. Scale bar = 5 mm. (**e**) Macroscopic wound area of *Flii*^+/−^, WT and *Flii*^Tg/Tg^ diabetic excisional wounds. Data displayed at mean ± SEM, *n* = 8. Statistical significance was calculated using a two-way ANOVA where * = *p* < 0.05, ** = *p* < 0.01, *** = *p* < 0.001, **** = *p* < 0.0001.

PDGFRβ/NG2 co-localisation was used to quantify pericyte numbers in unwounded skin of non-diabetic and diabetic *Flii*^+/−^, WT and *Flii*^Tg/Tg^ mice (Figure 3a). Flii expression negatively impacted pericyte presence in unwounded skin, with non-diabetic *Flii*^+/−^ and *Flii*^Tg/Tg^ skin displaying increased and decreased pericyte numbers respectively, when compared to WT mice (Figure 3b) (*n* = 8). In all three genotypes, a period of prolonged hyperglycaemia (>6 weeks) resulted in significant depletion of pericyte numbers in unwounded skin when compared to non-diabetic skin of the same genotype.

### 2.3. Increased Pericyte Numbers in Flii^+/−^ Diabetic Skin are Associated with an Increased Capacity for Healing

*Flii*^+/−^ wounds displayed an increase in the number of wound-resident PDGFRβ^+^/NG2^+^ pericytes (Figure 3c) (*n* = 8). In contrast, *Flii*^Tg/Tg^ wounds exhibited a diminished capacity for the recruitment of pericytes to the wound bed. These pericyte numbers correlated positively with diabetic healing rates. Macroscopically (Figure 3d), wound area decreased more rapidly in pericyte-rich Flii-attenuated wounds and was delayed in Flii overexpressing wounds (Figure 3e). Histological measurements of hematoxylin and eosin-stained sections (Supplementary Figure S1a) revealed that wound closure and re-epithelialisation displayed similar trends (Supplementary Figure S1b,c). 

### 2.4. Flii Attenuation and the Resultant Increase in Pericyte Numbers are Associated with Dampened Inflammation

**Figure 4:**
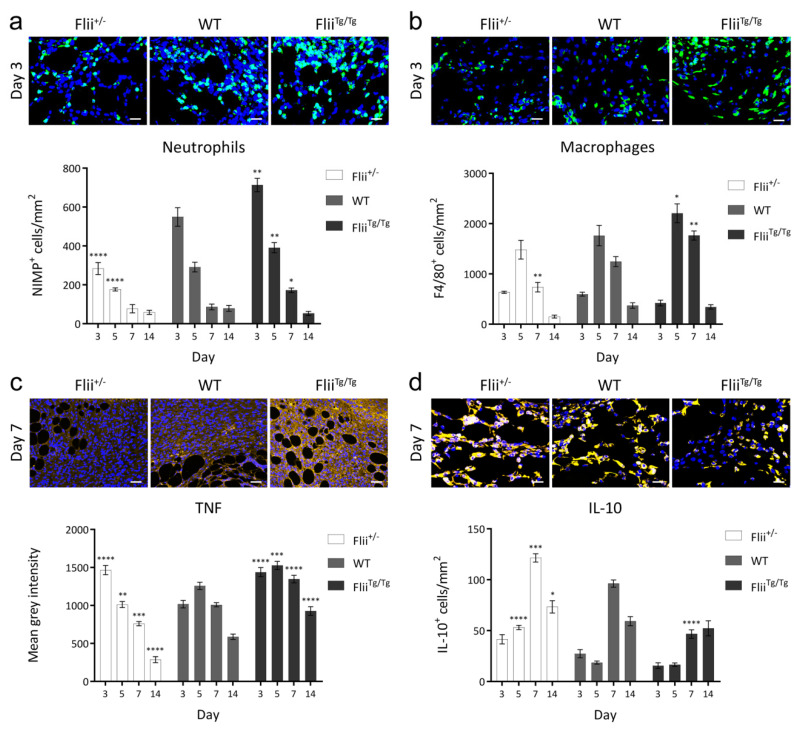
(**a**) Immunohistochemical detection and quantification of Anti-Neutrophil antibody (NIMP-R14) in 4 μm sections of paraffin-embedded diabetic *Flii*^+/−^, WT and *Flii*^Tg/Tg^ wounds. Images taken at 40x objective, scale bar = 20 μm. Data displayed as mean ± SEM, *n* = 7. (**b**) Immunohistochemical detection and quantification of F4/80 in 4 μm sections of paraffin-embedded diabetic *Flii*^+/−^, WT and *Flii*^Tg/Tg^ wounds. Images taken at 40× objective, scale bar = 20 μm. Data displayed as mean ± SEM, *n* = 7. (**c**) Immunohistochemical detection and quantification of tumour necrosis factor (TNF) in 4 μm sections of paraffin-embedded diabetic *Flii*^+/−^, WT and *Flii*^Tg/Tg^ wounds. Images taken at 20× objective, scale bar = 50 μm. Data displayed as mean ± SEM, *n* = 7. (**d**) Immunohistochemical detection and quantification of interleukin-10 (IL-10) in 4 μm sections of paraffin-embedded diabetic *Flii*^+/−^, WT and *Flii*^Tg/Tg^ wounds. Images taken at 40× objective, scale bar = 20 μm. Data displayed as mean ± SEM, *n* = 7. Statistical significance was calculated using a two-way ANOVA where * = *p* < 0.05, ** = *p* < 0.01, *** = *p* < 0.001, **** = *p* < 0.0001.

Anti-Neutrophil antibody (NIMP-R14) staining revealed that neutrophil infiltration was significantly decreased at days 3 and 5 of healing in pericyte-rich *Flii*^+/−^ wounds, while the low pericyte numbers of *Flii*^Tg/Tg^ wounds coincided with an exacerbated neutrophil presence in the wound bed at days 3, 5 and 7 (Figure 4a) (*n* = 7). Likewise, the presence of F4/80^+^ macrophages was decreased in *Flii*^+/−^ wounds at day 7 and increased in *Flii*^Tg/Tg^ wounds at days 5 and 7 (Figure 4b) (*n* = 7). The expression of pro-inflammatory cytokine tumour necrosis factor (TNF) in *Flii*^+/−^ wounds peaked at day 3 before rapidly resolving over the course of healing, while *Flii*^Tg/Tg^ wounds displayed prolonged and elevated TNF expression, which was not resolved by day 14 (Figure 4c). This, in conjunction with an increased abundance of anti-inflammatory interleukin-10 (IL-10)-expressing cells in *Flii*^+/−^ wounds (Figure 4d), suggests that Flii-attenuated, pericyte-rich wounds display a rapid and efficient period of inflammation when compared to their WT and *Flii*^Tg/Tg^ counterparts (*n* = 7). 

### 2.5. Flii Attenuation and the Resultant Increase in Pericyte Numbers are Associated with Enhanced Angiogenesis, Collagen Deposition and Remodelling

**Figure 5:**
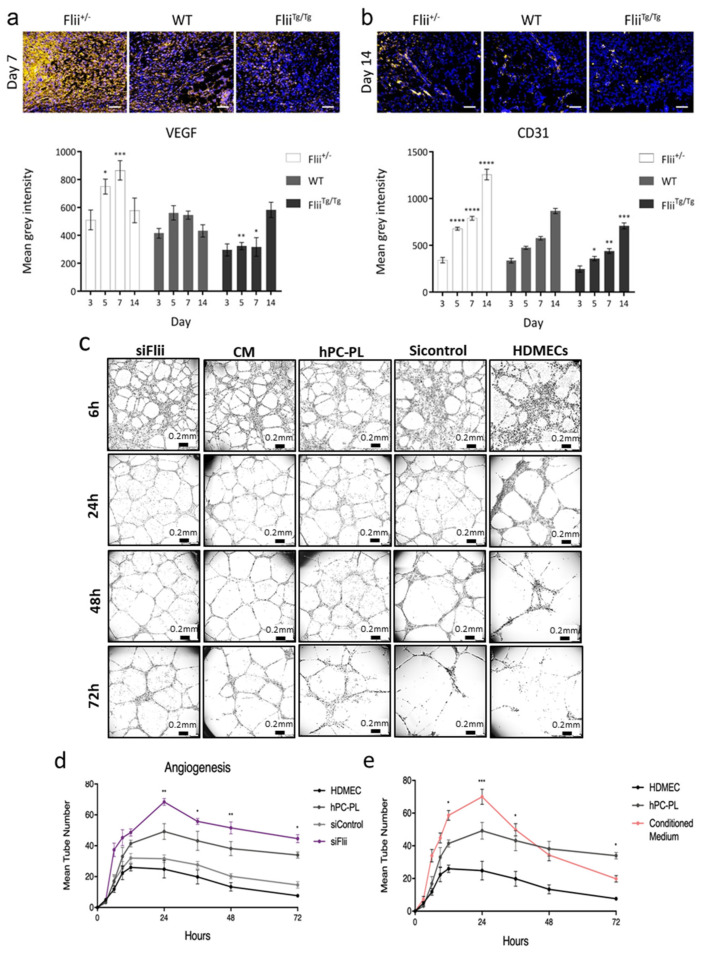
(**a**) Immunohistochemical detection and quantification of vascular endothelial growth factor (VEGF) in 4 μm sections of paraffin-embedded diabetic *Flii*^+/−^, WT and *Flii*^Tg/Tg^ wounds. Images taken at 20× objective, scale bar = 50 μm. Data displayed as mean ± SEM, *n* = 7. (**b**) Immunohistochemical detection and quantification of CD31 in 4 μm sections of paraffin-embedded diabetic *Flii*^+/−^, WT and *Flii*^Tg/Tg^ wounds. Images taken at 20× objective, scale bar = 50 μm. Data displayed as mean ± SEM, *n* = 7. (**c**) Representative images of tube formation in HDMECs alone and HDMECs co-cultured with hPC-PLs, siControl hPC-PLs, siFlii hPC-PLs and hPC-PL CM at 6, 24, 48 and 72 h. Images taken at 4× objective, scale bar = 200 μm. (**d**) Effect of untreated, siControl-treated or siFlii-treated hPC-PLs on human dermal microvascular endothelial cells (HDMECs) and (**e**) Effect of hPC-PL CM or hPC-PL co-culture on HDMEC sprouting in a matrigel tube formation assay. All data displayed as mean ± SEM, *n* = 5. Results representative of three independently conducted experiments. Statistical significance calculated by two-way ANOVA, where * = *p* < 0.05, ** = *p* < 0.01, *** = *p* < 0.001, **** = *p* < 0.0001.

The enhanced pericyte presence in *Flii*^+/−^ wounds corresponded with increased vascular endothelial growth factor (VEGF) signalling at days 5 and 7. In contrast, *Flii*^Tg/Tg^ wounds showed delayed upregulation of VEGF which did not increase until day 14 of healing (Figure 5a). Increased VEGF expression in *Flii*^+/−^ wounds was associated with enhanced revascularisation of the wound bed, as increased CD31 (endothelial cell marker) was observed in the tissue at days 5, 7 and 14. *Flii*^Tg/Tg^ mice displayed a decreased capacity to rebuild vascular structures, as revascularisation of the slow healing *Flii*^Tg/Tg^ wounds by day 14 only reached levels similar to those reached by the *Flii*^+/−^ wounds within the first week of healing (Figure 5b) (*n* = 7).

In vitro, human dermal microvascular endothelial cells (HDMECs) cultured with hPC-PLs formed increased numbers of tube-like structures with greater structural integrity than those formed by HDMECs alone. HDMECs co-cultured with siFlii hPC-PLs formed significantly increased numbers of tubes, and these structures lasted significantly longer in culture than those formed by HDMECs cultured with untreated or siControl-treated hPC-PLs (*n* = 5). Culturing of HDMECs alone in endothelial medium conditioned by hPC-PLs (hPC-PLs CM) (*n* = 6) led to increased tube formation, however these structures displayed more rapid degradation than any other treatment group (Figure 5c–e).

Collagen deposition and remodelling is important in the determination of scar formation after healing. Collagen I levels in *Flii*^+/−^ diabetic wounds were significantly decreased compared to WT wounds, and significantly increased in *Flii*^Tg/Tg^ wounds at day 14 (Supplementary Figure S2a). The level of collagen III was also increased in *Flii*^Tg/Tg^ wounds at day 14 (Supplementary Figure S2b), indicating more rapid deposition of collagens overall when Flii levels are increased.

## 3. Discussion

Pericyte depletion is documented in a number of diabetic tissues, and the low numbers of these important cells in the dermis of the skin may adversely affect the regulation of inflammation and vascular processes. Staining for specific markers of pericytes revealed that diabetic wounds contained significantly fewer pericytes in the dermis than acute wounds from healthy patients. Diabetic wounds also exhibited increased expression of Flii, a known negative regulator of cutaneous healing. This indicates an inverse relationship between Flii expression and pericyte presence and suggests a role for Flii in the regulation of pericyte behaviour. In an inducible murine model of type 1 diabetes carried out in mice expressing differential levels of Flii expression, pericyte numbers were highest in *Flii*^+/−^ wounds and lowest in *Flii*^Tg/Tg^ wounds when compared to WT, reiterating the inverse relationship between Flii expression and pericyte numbers observed in human samples.

Flii attenuation in vitro led to increased proliferation and migration of pericytes. This may explain the increased pericyte numbers found residing basally in *Flii*^+/−^ skin and the apparent enhanced capacity of *Flii*^+/−^ skin to recruit pericytes to wounds during healing. The increased pericyte numbers in Flii-attenuated skin were also associated with an enhanced capacity for healing. Zhuang et al. [16] have previously demonstrated that pericytes in the dermis encourage basal keratinocytes, via BMP-2 signalling, to undergo increased planar cell divisions leading to faster maturation of the epithelial layer. While re-epithelialisation was only mildly accelerated in *Flii*^+/−^ wounds, this is consistent with the increased pericyte numbers observed in the dermis of these mice. 

Pericyte-rich *Flii*^+/−^ wounds displayed dampened infiltration of neutrophils, while *Flii*^Tg/Tg^ wounds exhibited a heightened and prolonged neutrophil response. This amplified neutrophil infiltration in *Flii*^Tg/Tg^ wounds is reminiscent of the elevated neutrophil infiltrate often observed in human chronic wounds. The cytoskeletal relaxation of pericytes on the abluminal surface of blood vessels is required for the opening of gaps termed ‘low expression regions’ (LERs) between pericytes, which allow for neutrophil passage through the endothelial and sub-endothelial layers of the vessel wall [17]. This pericyte relaxation is mediated by Rho/ROCK signalling, whereby neutrophils cause inhibition of the Rho/ROCK pathway in pericytes, leading to suppression of actomyosin-based contractility [18]. It has been shown that Flii does not modulate Rho/ROCK signalling, but does regulate actin cytoskeletal remodelling through a Rac-1-dependent pathway [19]. Therefore, dampened neutrophil infiltration in *Flii*^+/−^ wounds may be two-fold: there are more pericytes present in these wounds to cover the abluminal surface of vessels and prevent vascular leakage, and Flii attenuation in those pericytes may additionally impact neutrophil infiltration by altering pericyte contractility.

Macrophage recruitment was also diminished in Flii-attenuated wounds. This may be partly due to the decreased presence of neutrophils in the granulation tissue which release chemokines to attract macrophages [20]. In Flii overexpressing wounds, macrophage infiltration was aggravated, echoing the exacerbated inflammatory status of human chronic wounds. Flii expression correlated with an inflammatory cytokine profile within the diabetic wounds, as increased IL-10-expressing cells were present in *Flii*^+/−^ wounds. This coincided with rapidly resolving TNF expression, while in contrast, *Flii*^Tg/Tg^ wounds displayed delayed upregulation of TNF which did not resolve within 14 days. 

In models of inducible diabetes, both TNF-deficient mice and mice treated with a TNF inhibitor exhibit significantly less pericyte apoptosis than control diabetic mice, implicating TNF expression in mechanisms of pericyte depletion [21]. Decreased pericyte numbers in *Flii*^Tg/Tg^ wounds were associated with a significant upregulation of TNF expression within the wound bed, which may explain pericyte depletion in diabetic wounds with high Flii expression. TNF is a potent neutrophil chemoattractant, facilitating neutrophil extravasion by signalling for the upregulation of adhesion molecules on endothelial cells [22]. This may also contribute to the heightened neutrophil extravasion observed in *Flii*^Tg/Tg^ wounds. 

Increased pericyte numbers in *Flii*^+/−^ wounds were associated with an enhanced capacity for revascularisation of the wounded tissue after injury. This was demonstrated by increased CD31 expression in *Flii*^+/−^ wounds and was mirrored by significantly decreased expression in *Flii*^Tg/Tg^ wounds. These changes were accompanied by upregulation and downregulation of VEGF signalling within *Flii*^+/−^ and *Flii*^Tg/Tg^ wounds, respectively. Co-culture of mesenchymal and endothelial cells induces differentiation of the mesenchymal cells to a pericyte-like phenotype and causes upregulation of VEGF expression [23]. Pericyte-derived VEGF has therefore been suggested to mediate the endothelial proliferation and migration necessary for neo-angiogenesis and endothelial cell survival. This is consistent with the increased VEGF levels observed in pericyte-rich *Flii*^+/−^ diabetic wounds.

Pericyte regulation of endothelial function is both physical and paracrine. In vitro formation of angiogenic “tubes” by endothelial cells was increased in the presence of pericytes, and these pericyte-supported tubes displayed enhanced stability. When Flii was attenuated in pericytes, significantly more tubes formed and these structures degraded less rapidly, confirming that Flii negatively effects pericyte contributions to angiogenesis. While the presence of pericyte-conditioned medium was sufficient to enhance early endothelial sprouting, these vessels collapsed quickly, illustrating that the physical presence of pericytes is necessary to provide support and longevity. 

## 4. Materials and Methods

### 4.1. Human Samples

The collection of human skin samples was approved by the Human Research Ethics Committee (TQEH/LMH/MH) (HREC/12/TQEHLMH/107/EXT01 Approved 30/5/2014) and was carried out in accordance with the Declaration of Helsinki (1964). Prior to the collection of tissue samples, informed consent was obtained from each patient. After an application of local anaesthetic, one 6 mm wound biopsy was taken from around the edge of the wound of 6 patients with diabetic ulcers (ulcer duration ≥ 6 weeks; 4 men and 2 women with a mean age of 64 years) and 6 non-diabetic patients with acute wounds (wound duration ≤ 4 weeks; 4 women and 2 men with a mean age of 40 years). Wound samples were fixed in formalin and processed for immunohistological assessment.

### 4.2. Animal Studies

All transgenic mice were sourced from an in-house breeding colony, under approval from the Women’s and Children’s Health Network Animal Ethics Committee (AE1055/11/19 Approved 06/12/2016). All mice were congenic on a BALB/c background. Flii overexpressing mice, which are homozygous for a human Flii transgene in addition to the two endogenous copies of murine Flii, were designated “*Flii*^Tg/Tg^”. The generation of these mice has been previously described [24]. Mice with a heterozygous *Flii* knockout were designated “*Flii*^+/−^”. The generation of this strain has also been previously described [25]. Wild-type litter mates from this line (designated “WT”) were used as controls for both strains.

### 4.3. Murine Model of Inducible Diabetes

All animal procedures were approved by the Women’s and Children’s Health Network Animal Ethics Committee (AE1021/10/2018 Approved 14/10/2018) and were carried out in accordance with the Australian Code for the care and use of animals for scientific purposes. Diabetes was induced in 12-week-old female *Flii*^+/−^, WT and *Flii*^Tg/Tg^ mice with body weights of 18–22 g by STZ (Sigma-Aldrich, Darmstadt, Germany) intraperitoneal injection, as described previously [14], and in more detail in the Appendix A. Diabetes was confirmed by verification of a consistently elevated (>15.25 mg/L) blood glucose level (BGL) for a sustained period of >6 weeks. A 50% rate of successful diabetic induction was expected based on the literature and previous trials conducted. This was observed in the WT cohort (53% induction), however *Flii*^+/−^ mice exhibited an increased susceptibility to induction (76% induction), while *Flii*^Tg/Tg^ mice displayed a decreased susceptibility to the development of diabetes in response to STZ treatment (39% induction). Mice that were confirmed to be diabetic were subjected to a model of excisional punch biopsy as described previously [26] and wounds were collected 3, 5, 7 or 14 days post-surgery. Wound samples were fixed in 10% neutral buffered formalin overnight, transferred to 70% ethanol for 24 h, embedded in paraffin wax and sectioned for analysis. 

### 4.4. Histological and Immunohistochemistry Assessment of Wounds

4 μm wound sections were dewaxed in xylene and brought to water through ethanol to allow staining with Hematoxylin and Eosin, as described previously [27]. Wound sections were dewaxed in xylene and brought to water in ethanol for immunohistochemical staining, as described previously [28]. Tissue sections were blocked in 3% blocking serum for 30 min at room temperature and incubated with Flightless I (1 µg/mL, sc-21716, Santa Cruz Biotechnology, Dallas, TX, USA), β-tubulin (10 µg/mL, T8328, Sigma-Aldrich, Castle Hill, NSW, Australia), NG2 (5 µg/mL, AB5320, Merck, Bayswater, VIC, Australia), PDGFRβ (1 µg/mL, AF1042, R&D Systems, Minneapolis, MN, USA), collagen I (5 µg/mL, 600-401-103-0.5, Rockland Immunochemicals, Gilbertsville, PA, USA), collagen III (5 µg/mL, 600-401-105-0.5, Rockland Immunochemicals, Gilbertsville, PA, USA), NIMP-R14 (0.5 µg/mL, sc-59338, Santa Cruz Biotechnology, Dallas, TX, USA), F4/80 (5 µg/mL, MCA4975, Bio-Rad, Gladesville, NSW, Australia), TNF (0.5 µg/mL, sc-52746, Santa Cruz Biotechnology, Dallas, TX, USA), IL-10 (2.5 µg/mL, ab9969, Abcam, Cambridge, UK), CD31 (0.2 µg/mL, ab28364, Abcam, Cambridge, UK) or VEGF (5 µg/mL, ab46154, Abcam, Cambridge, UK) antibodies overnight at 4 °C. Tissue sections were subsequently incubated with secondary goat anti-rabbit Alexa Fluor 594 (A11011), goat anti-rat Alexa Fluor 488 (A11006), goat anti-mouse Alexa Fluor 633 (A21050), goat anti-mouse Alexa Fluor 594 (A11004), goat anti-mouse Alexa Fluor 488 (A11006) or donkey anti-goat Alexa Fluor 633 (A21082) (5 µg/mL, Invitrogen, Carlsbad, CA, USA). Image analysis was performed using CellSens Imaging Software to measure either positive cells per area or mean fluorescence intensity, which was automatically calculated as intensity per area.

### 4.5. hPC-PL Culture and siFlii Knockdown

Human pericytes from placenta (hPC-PL) were purchased and cultured in Human Pericyte Media as per company instructions (Promocell, Heidelberg, Germany). Transfection of siFlii (GCUGGAACACUUGUCUGUGdTdT, CACAGACAAGUGUUCCAGCdTdT) or siControl (GGUUAGCCGCACGUUAGUUdTdT, AACUAACGUGCGGCUAACCdTdT) constructs (Sigma-Aldrich, Darmstadt, Germany) was carried out in suspension using Lipofectamine 2000 (ThermoFisher Scientific, Waltham, MA, USA), as described previously [29].

### 4.6. Immunocytochemistry

Untreated, siFlii-treated and siControl-treated hPC-PL were seeded at 1 × 10^4^ cells/well in a black 96-well plate (Corning, NY, USA) and incubated for 48 h. Cells were fixed in ice-cold methanol, permeabilised with 0.5% Tween20, blocked with 3% normal goat serum and incubated with primary antibody (Flightless I and β-tubulin) for 2 h at room temperature. Cells were washed and incubated with secondary antibody for 1 h, stained for 5 min with 1:5000 DAPI (4′,6-diamidino-2-phenylindole), retained in phosphate buffered saline (PBS) and imaged using CellSens software. Results were representative of three independent experiments with six technical replicates per experiment.

### 4.7. Proliferation

24 h after knockdown, untreated, siFlii-treated and siControl-treated hPC-PL were seeded at 5 × 10^3^ cells/well in a 96-well plate and allowed to adhere. After 6 h, 15 µl WST1 reagent (Sigma-Aldrich, Darmstadt, Germany) was added to each well and incubated for 30 min at 37 °C. Absorbance at 620 nm was read and subtracted from absorbance at 460 nm, after which the plate was discarded. WST1 incubation and absorbance measurements were repeated on duplicate plates at 24 and 48 h. Results are representative of three independent experiments with six technical replicates per experiment.

### 4.8. Migration

12 h after knockdown, untreated, siFlii-treated and siControl-treated hPC-PL were seeded at 2 × 10^4^ cells/well in a 96-well plate and incubated for 12 h. Wells were “scratched” with a WoundMaker™ (Essen BioScience, Ann Arbor, MI, USA), washed once with PBS and the media was replaced. Scratches were imaged at 0, 3, 6, 9, 12, 18, 24 and 48 h and the mean scratch width for each group was calculated using CellSens software. Results were representative of three independent experiments with six technical replicates per experiment.

### 4.9. Angiogenesis

24 h after knockdown, untreated, siFlii-treated and siControl-treated hPC-PL were seeded in reduced growth factor matrigel (Corning, NY, USA) in a 15-well angiogenesis plate (ibidi GmbH, Bavaria, Germany) with human dermal microvascular endothelial cells (HDMECs) (Promocell, Heidelberg, Germany) at a ratio of 1:10. Cells were imaged at 0, 3, 6, 9, 12, 24, 36, 48 and 72 h and blinded analysis of the number of tubes formed was calculated for each well using CellSens software. Results are representative of three independent experiments with five technical replicates per experiment.

### 4.10. Western Blotting

Western blot analysis of Flii protein expression was carried out as described previously [28] on hPC-PL cell lysates 48 h after siRNA treatment. Protein samples were made up to 15 uL and 3 uL of 5× SDS PAGE loading buffer (25 nM Tris pH 6.8, 8% glycerol, 1% SDS, 0.02% bromophenol blue, 5% 2-mercaptoethanol) was added to each sample. Samples were boiled for 5 min at 100 °C to denature, condensation was centrifuged, and samples were vortexed immediately prior to loading. Prepared lysates were loaded at 15 μL/1.5 mm well in a polyacrylamide gel of appropriate percentage (10% sodium dodecyl sulfate-polyacrylamide gel electrophoresis (SDS-PAGE)) for the resolution and visualisation of Flightless I protein. Electrophoresis was performed in 1× Western Running Buffer at 25 mA per gel until the dye front approached the bottom of the gel. Transfer sponges, Whatman paper and nitrocellulose membrane (BioTraceTM NT, Pall Corporation, FL, USA) were soaked in cold 1× Western Transfer Buffer with 20% methanol and assembled with the gel into a transfer cassette. Transfer was carried out at 250 mA for 1 h at 4 °C. The membrane was blocked in 15% skim milk for 1 h and blotted with primary antibodies against Flii (SC30046, 1 µg/mL Santa Cruz Biotechnologies, Dallas, TX, USA) and β-tubulin (T8328, 0.4 µg/mL, Sigma-Aldrich, Castle Hill, NSW, Australia) in 6% skim milk overnight at 4 °C. The membrane was washed 3 times for 5 min in 1× TBST (TBS, 0.1% Tween20) and probed with secondary antibodies (DAKO P0447 1:2000 and P0448 1:2000, Agilent, Santa Clara CA, USA) for 1 h at room temperature. The membrane was washed 2 times for 5 min in 1× TBST, once for 5 min in TBS, developed using a Clarity Western ECL kit (Bio-Rad, Gladesville, NSW, Australia), and imaged under UV light using a SynGene G-Box. Results were representative of three independent experiments.

### 4.11. Statistical Analysis

All analysis was carried out in Graphpad Prism (Graphpad, CA, USA). For histological and immunohistochemical comparisons between *Flii*^+/−^, WT and *Flii*^Tg/Tg^ wounds, statistical significance was calculated using a 2-way analysis of variance (ANOVA) with multiple comparisons. For in vitro analyses, statistical significance between treatment groups was calculated using a 2-way ANOVA with multiple comparisons. Annotation of significance above any time point indicates statistical significance between the untreated and siFlii groups at the time point. A *p*-value of <0.05 was considered significant. All data are displayed as mean ± SEM.

## 5. Conclusions

Understanding the mechanisms of diabetic repair will be important in the development of improved therapies to enhance the healing of DFUs. Pericytes are important for the regulation of many processes commonly dysregulated in diabetic wounds. The data presented here indicates that Flii regulates pericyte presence and function in diabetic wounds, and illuminates an additional mechanism by which Flii attenuation can enhance healing outcomes in both acute and diabetic wounds. 

## Supplementary Materials

Supplementary materials can be found at https://www.mdpi.com/1422-0067/21/16/5599/s1. Figure S1 (a) Hematoxylin and Eosin staining of representative *Flii*^+/−^, WT and *Flii*^Tg/Tg^ diabetic excisional day 3, 5, 7 and 14 wounds. Wound width indicated by dashed white line. Composite images taken at 10× objective, scale bar = 0.5 m. (b) Histological wound width measurements of *Flii*^+/−^, WT and *Flii*^Tg/Tg^ diabetic excisional day 3, 5, 7 and 14 wounds. Data displayed as mean ± SEM, *n* = 8. (c) Histological measurement of re-epithelialisation represented as % of total epithelial length. Data displayed as mean ± SEM, *n* = 8. Statistical significance was calculated using a two-way ANOVA where * = *p* < 0.05, ** = *p* < 0.01, *** = *p* < 0.001, **** = *p* < 0.0001. Figure S2. (a) Immunohistochemical detection and quantification of collagen I in 4 μm sections of paraffin-embedded diabetic *Flii*^+/−^, WT and *Flii*^Tg/Tg^ wounds. (**b**) Immunohistochemical detection and quantification of collagen III in 4 μm sections of paraffin-embedded diabetic *Flii*^+/−^, WT and *Flii*^Tg/Tg^ wounds. Images taken at 20× objective, scale bar = 50 μm. Data displayed as mean ± SEM, *n* = 7. Statistical significance was calculated using a two-way ANOVA where * = *p* < 0.05, ** = *p* < 0.01, *** = *p* < 0.001, **** = *p* < 0.0001.

## Abbreviations

BGL	Blood glucose level
DFUs	Diabetic foot ulcers
Flii	Flightless I
Flii^+/−^	Flightless I heterozygous knockout
Flii^Tg/Tg^	Flightless I transgenic overexpressing
FnAb	Flii neutralizing antibody
HDMEC	Human dermal microvascular endothelial cells
hPC-PL	Human Pericytes from Placenta
IL-10	Interleukin-10
LER	Low expression regions
LRR	Leucine-rich repeat region
MSC	Mesenchymal stem cell
NG2	Neural glial antigen 2
PDGFRβ	Platelet-derived growth factor receptor beta
STZ	Streptozotocin
TNF	Tumour necrosis factor
VEGF	Vascular endothelial growth factor

## Appendix A. Animal Studies

### Appendix A.1. Sample Size Determination

The sample size for the mouse studies was calculated using G power. A reduction in surface area of 20% or more was considered biologically significant. Power studies for the diabetic mouse model showed that a sample size of 8 would give 90% power using a 5% test level and a one-tailed test.

### Appendix A.2. Murine Model of Inducible Diabetes

Prior to injection, mice were fasted for 4 h, after which each mouse received an intraperitoneal injection of a previously optimised low dose of streptozocin (STZ) (50 mg/kg) for 5 consecutive days [14]. Non-diabetic control mice were similarly fasted and received equivalent injections of vehicle only to serve as a control. Following the commencement of injections, mice were observed twice daily and weighed daily for the first 3 days, after which observation continued once daily. Tail vein sampling was used to monitor non-fasting blood glucose levels (BGL) every 2–3 days for 4 weeks following the culmination of STZ injections. After these 4 weeks had elapsed, animals with confirmed diabetes (as indicated by a BGL of greater than 15 mmol/L) were administered a subcutaneous injection of insulin (2 IU) every second day to maintain body weight and prevent ketoacidosis. Any animals which failed to maintain a weight within 10% of their starting weight or display BGL elevated above 25 mmol/L received an increased dose of 4 IU insulin every second day. Mice were maintained on this regimen for a further 2 weeks, after which mice with confirmed hyperglycaemia (BGL greater than 15 mmol/L) were subjected to the excisional wound model, while mice failing to display hyperglycaemia were excluded from progressing further in the study at this point. In keeping with the three Rs (Replacement, Refinement and Reduction), and in preference to the induction of new mice, previously induced mice which did not exhibit hyperglycaemia after 6 weeks received a ‘re-induction’ protocol of 50 mg/kg STZ IP injection for a further 5 days. The induction rates following re-induction were significantly elevated in all genotypes.

### Appendix A.3. Murine Model of Excisional Wound Healing

In accordance with WCH animal ethics approval (AE1021/10/18), diabetic *Flii*^+/−^, WT and *Flii*^Tg/Tg^ mice were subjected to a model of excisional wound healing. Prior to wound induction, diabetic mice were housed in smooth-bottom plastic cages with paper bedding and free access to water and rodent food pellets (22 °C and 12 h dark/light cycle). Environment enrichments were provided by adding clean toilet rolls and toys to the cages to prevent stress and boredom. Mice were anaesthetised by inhalation of 5% isoflurane (Abbott Australasia, NSW, Australia) and 5% oxygen at 2 L/min for induction and maintained at 2% isoflurane with 2% oxygen at 700 mL/min of maintenance. Prior to surgery, analgesia (Buprenorphine at 0.05 mg/kg, MSD, NSW, Australia) was administered to each mouse. The dorsal surface of the mice was shaved and depilated. Six mm punch biopsies (EBOS, NSW, Australia) were used to create two 6 mm^2^ full-thickness excisional wounds on the dorsal surface of the mice 5 mm either side of the midline and 10 mm from the base of the skull. Mice were allowed to recover from anaesthesia on a heated pad, after which they were returned to individual cages and allowed to recover in a warm, dry cage with water and food ad libitum. Wounds were collected 3, 5, 7 or 14 days post-surgery and wound samples were fixed in 10% neutral buffered formalin overnight, transferred to 70% ethanol for 24 h, embedded in paraffin wax and sectioned for analysis (*n* = 8 mice, 16 wounds for each genotype at each timepoint).

## References

[1] Rask-Madsen C., King G.L. (2013). Vascular complications of diabetes: Mechanisms of injury and protective factors. Cell Metab..

[2] Boulton A.J., Vileikyte L., Ragnarson-Tennvall G., Apelqvist J. (2005). The global burden of diabetic foot disease. Lancet.

[3] Dinh T., Tecilazich F., Kafanas A., Doupis J., Gnardellis C., Leal E., Tellechea A., Pradhan L., Lyons T.E., Giurini J.M. (2012). Mechanisms involved in the development and healing of diabetic foot ulceration. Diabetes.

[4] Thomas H., Cowin A., Mills S. (2017). The Importance of Pericytes in Healing: Wounds and other Pathologies. Int. J. Mol. Sci..

[5] Mills S.J., Cowin A.J., Kaur P. (2013). Pericytes, mesenchymal stem cells and the wound healing process. Cells.

[6] Beltramo E., Porta M. (2013). Pericyte Loss in Diabetic Retinopathy: Mechanisms and Consequences. Curr. Med. Chem..

[7] Tilton R.G., Faller A.M., Burkhardt J.K., Hoffmann P.L., Kilo C., Williamson J.R. (1985). Pericyte degeneration and acellular capillaries are increased in the feet of human diabetic patients. Diabetologia.

[8] Archer S.K., Behm C.A., Claudianos C., Campbell H.D. (2004). The flightless I protein and the gelsolin family in nuclear hormone receptor-mediated signalling. Biochem. Soc. Trans..

[9] Lee Y.H., Campbell H.D., Stallcup M.R. (2004). Developmentally essential protein flightless I is a nuclear receptor coactivator with actin binding activity. Mol. Cell. Biol..

[10] Silacci P., Mazzolai L., Gauci C., Stergiopulos N., Yin H., Hayoz D. (2004). Gelsolin superfamily proteins: Key regulators of cellular functions. Cell. Mol. Life Sci. CMLS.

[11] Kopecki Z., Cowin A. (2008). Flightless I: An actin-remodelling protein and an important negative regulator of wound repair. Int. J. Biochem. Cell Biol..

[12] Cowin A.J., Adams D.H., Strudwick X.L., Chan H., Hooper J.A., Sander G.R., Rayner T.E., Matthaei K.I., Powell B.C., Campbell H.D. (2007). Flightless I deficiency enhances wound repair by increasing cell migration and proliferation. J. Pathol..

[13] Jackson J.E., Kopecki Z., Adams D.H., Cowin A.J. (2012). Flii neutralizing antibodies improve wound healing in porcine preclinical studies. Wound Repair Regen..

[14] Ruzehaji N., Kopecki Z., Melville E., Appleby S.L., Bonder C.S., Arkell R.M., Fitridge R., Cowin A.J. (2014). Attenuation of flightless I improves wound healing and enhances angiogenesis in a murine model of type 1 diabetes. Diabetologia.

[15] Wu J., Yan L.-J. (2015). Streptozotocin-induced type 1 diabetes in rodents as a model for studying mitochondrial mechanisms of diabetic β cell glucotoxicity. Diabetes Metab. Syndr. Obes..

[16] Zhuang L., Lawlor K.T., Schlueter H., Pieterse Z., Yu Y., Kaur P. (2018). Pericytes promote skin regeneration by inducing epidermal cell polarity and planar cell divisions. Life Sci. Alliance.

[17] Proebstl D., Voisin M.-B., Woodfin A., Whiteford J., D’Acquisto F., Jones G.E., Rowe D., Nourshargh S. (2012). Pericytes support neutrophil subendothelial cell crawling and breaching of venular walls in vivo. J. Exp. Med..

[18] Wang S., Cao C., Chen Z., Bankaitis V., Tzima E., Sheibani N., Burridge K. (2012). Pericytes Regulate Vascular Basement Membrane Remodeling and Govern Neutrophil Extravasation during Inflammation. PLoS ONE.

[19] Kopecki Z., O’Neill G.M., Arkell R.M., Cowin A.J. (2011). Regulation of focal adhesions by flightless i involves inhibition of paxillin phosphorylation via a Rac1-dependent pathway. J. Investig. Dermatol..

[20] Amulic B., Cazalet C., Hayes G.L., Metzler K.D., Zychlinsky A. (2012). Neutrophil Function: From Mechanisms to Disease. Annu. Rev. Immunol..

[21] Joussen A.M., Doehmen S., Le M.L., Koizumi K., Radetzky S., Krohne T.U., Poulaki V., Semkova I., Kociok N. (2009). TNF-alpha mediated apoptosis plays an important role in the development of early diabetic retinopathy and long-term histopathological alterations. Mol. Vis..

[22] De Vries I.J., Langeveld-Wildschut E.G., van Reijsen F.C., Dubois G.R., van den Hoek J.A., Bihari I.C., van Wichen D., de Weger R.A., Knol E.F., Thepen T. (1998). Adhesion molecule expression on skin endothelia in atopic dermatitis: Effects of TNF-alpha and IL-4. J. Allergy Clin. Immunol..

[23] Darland D.C., Massingham L.J., Smith S.R., Piek E., Saint-Geniez M., D’Amore P.A. (2003). Pericyte production of cell-associated VEGF is differentiation-dependent and is associated with endothelial survival. Dev. Biol..

[24] Thomsen N., Chappell A., Ali R.G., Jones T., Adams D.H., Matthaei K.I., Campbell H.D., Cowin A.J., Arkell R.M. (2011). Mouse strains for the ubiquitous or conditional overexpression of the Flii gene. Genesis.

[25] Campbell H.D., Fountain S., McLennan I.S., Berven L.A., Crouch M.F., Davy D.A., Hooper J.A., Waterford K., Chen K.S., Lupski J.R. (2002). Fliih, a gelsolin-related cytoskeletal regulator essential for early mammalian embryonic development. Mol. Cell. Biol..

[26] Ruzehaji N., Mills S.J., Melville E., Arkell R., Fitridge R., Cowin A.J. (2013). The influence of Flightless I on Toll-like-receptor-mediated inflammation in a murine model of diabetic wound healing. BioMed Res. Int..

[27] Strudwick X.L., Waters J.M., Cowin A.J. (2017). Flightless I Expression Enhances Murine Claw Regeneration Following Digit Amputation. J. Investig. Dermatol..

[28] Kopecki Z., Yang G.N., Jackson J.E., Melville E.L., Calley M.P., Murrell D.F., Darby I.A., O’Toole E.A., Samuel M.S., Cowin A.J. (2015). Cytoskeletal protein Flightless I inhibits apoptosis, enhances tumor cell invasion and promotes cutaneous squamous cell carcinoma progression. Oncotarget.

[29] Martens P.J., Ly M., Adams D.H., Penzkover K.R., Strudwick X., Cowin A.J., Poole-Warren L.A. (2015). In vivo delivery of functional Flightless I siRNA using layer-by-layer polymer surface modification. J. Biomater. Appl..

