# Supplementary material for: Attenuation of Flightless I Increases Human Pericyte Proliferation, Migration and Angiogenic Functions and Improves Healing in Murine Diabetic Wounds
Source: Int J Mol Sci. 2020 Aug 5;21(16):5599. doi: 10.3390/ijms21165599 (PMC7460558; doi:10.3390/ijms21165599)
Supplement: Supplementary file 1 [file ijms-21-05599-s001.pdf]

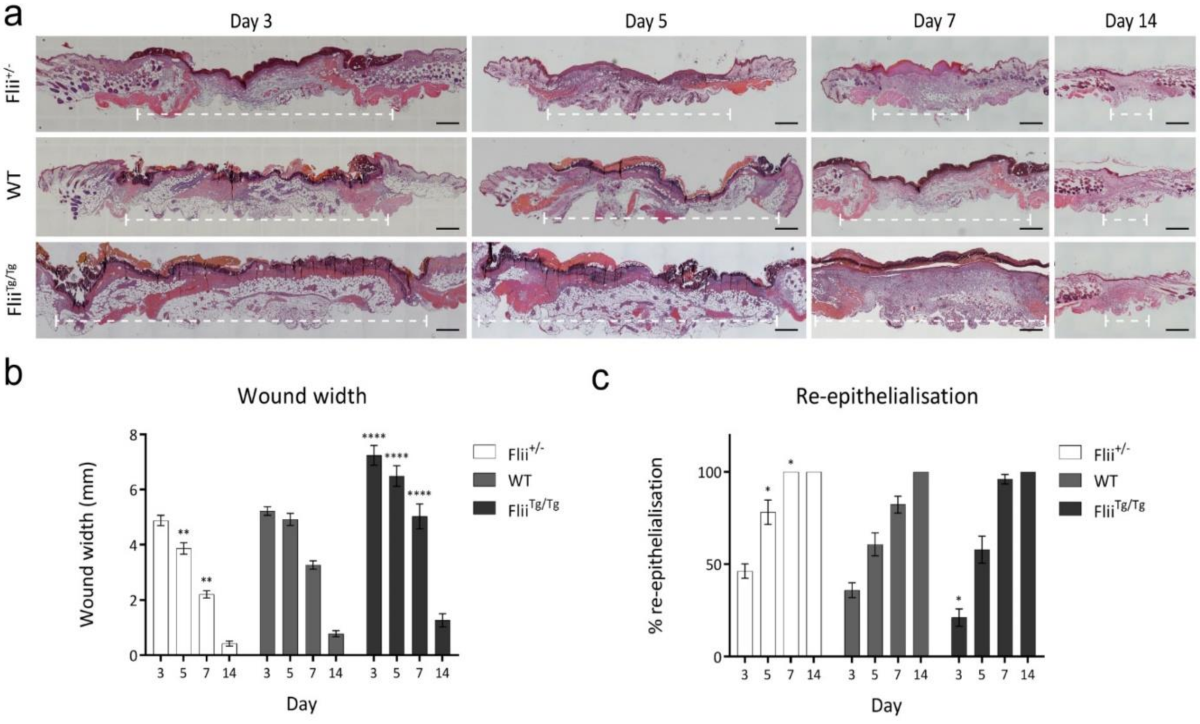

Figure S1 (**a**) Hematoxylin and Eosin staining of representative Flii$^{+/-}$, WT and Flii$^{Tg/Tg}$ diabetic excisional day 3, 5, 7 and 14 wounds. Wound width indicated by dashed white line. Composite images taken at 10× objective, scale bar = 0.5 m. (**b**) Histological wound width measurements of Flii$^{+/-}$, WT and Flii$^{Tg/Tg}$ diabetic excisional day 3, 5, 7 and 14 wounds. Data displayed as mean ± SEM, n = 8. (**c**) Histological measurement of re-epithelialisation represented as % of total epithelial length. Data displayed as mean ± SEM, n = 8. Statistical significance was calculated using a two-way ANOVA where * = $p < 0.05$, ** = $p < 0.01$, *** = $p < 0.001$, **** = $p < 0.0001$.

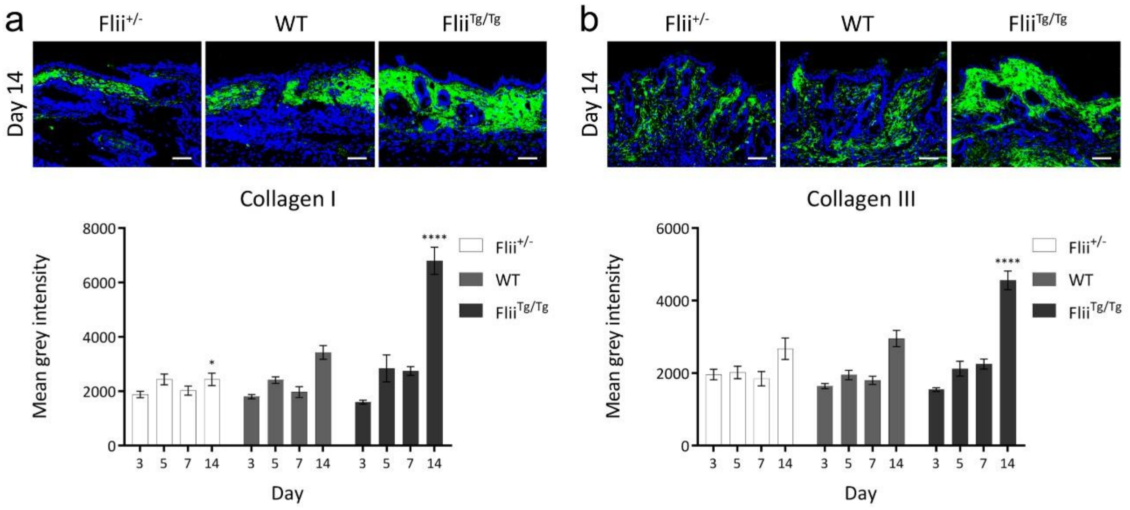

Figure S2. (**a**) Immunohistochemical detection and quantification of collagen I in 4 μm sections of paraffin-embedded diabetic Flii$^{+/-}$, WT and Flii$^{Tg/Tg}$ wounds. (**b**) Immunohistochemical detection and quantification of collagen III in 4 μm sections of paraffin-embedded diabetic Flii$^{+/-}$, WT and Flii$^{Tg/Tg}$ wounds. Images taken at 20× objective, scale bar = 50 μm. Data displayed as mean ± SEM, n = 7.

Statistical significance was calculated using a two-way ANOVA where * = $p < 0.05$, ** = $p < 0.01$, *** = $p < 0.001$, **** = $p < 0.0001$.
